# Racial differences in 5-year relative survival rates of cervical cancer by stage at diagnosis, between African American (black) and white women, living in the state of Alabama, USA

**DOI:** 10.1186/s12885-020-07338-7

**Published:** 2020-09-01

**Authors:** Ehsan Abdalla, Roberta Troy, Souleymane Fall, Isra Elhussin, Oyoyo Egiebor-Aiwan, David Nganwa

**Affiliations:** 1 Department of Graduate Public Health (College of Veterinary Medicine), Tuskegee University, 1200 W Montgomery Rd, Tuskegee, AL 36088 USA; 2 Biology Department (College of Arts and Sciences), Tuskegee University, 1200 W Montgomery Rd, Tuskegee, AL 36088 USA; 3 Department of Agriculture and Environmental Sciences (College of Agriculture, Environment and Nutrition Sciences), Tuskegee University, 1200 W Montgomery Rd, Tuskegee, AL 36088 USA; 4 Integrative Biosciences PhD program, Tuskegee University, 1200 W Montgomery Rd, Tuskegee, AL 36088 USA; 5 Southern Illinois School of Medicine, 2711 Covered Wagon, Trail Springfield, IL 62711 USA; 6 Department of Pathobiology/Department of Graduate Public Health, Tuskegee University, 1200 W Montgomery Rd, Tuskegee, AL 36088 USA

**Keywords:** Cervical cancer, 5-year relative survival ratios (RSRs), Urban, Black Belt and other rural counties of Alabama

## Abstract

**Background:**

Our objective was to assess racial differences in the 5-year relative survival rates (RSRs) of Cervical Cancer (CerCancer) by stage at diagnosis, between Black and White women, living in Alabama, USA.

**Methods:**

Data for 3484 Blacks and 21,059 Whites diagnosed with CerCancer were extracted from the 2004 to 2013 Surveillance, Epidemiology, and End Results (SEER) database. We incorporated age groups, CerCancer stages, county, and year of diagnosis to compare the RSR between Blacks and Whites, using SEER*Stat software.

**Results:**

In urban, Black Belt (BB) and other rural counties, Whites diagnosed with localized stage of CerCancer always had better chances of survival because their RSRs were always more than 77%, compared to Blacks. Only exception was in Blacks living in other rural counties, who had a significantly higher RSR of 83.8% (95% Cl, 74.2–90.1). Which was the same as in Whites (83.8% (95% CI 74.5–89.9) living in BBC. Although, in other rural counties, Whites had a slightly lower RSR of 83.7% (95% CI 79.9–86.8%), their RSR was better compared to Blacks and Whites living in BB and other rural counties who had slightly higher RSRs of 83.8%. This was due to statistical precision, which depended on their larger sample size and a lower variability therefore, more reliability resulting in a tighter confidence interval with a smaller margin of error. In all the three county groups, Whites 15–44 years old diagnosed with localized stage of CerCancer had a higher RSR of 93.6% (95% CI 91.4–95.2%) for those living in urban and BB counties, and 94.6% (95% CI 93.6–95.4) for those living in other rural counties. The only exception was in Blacks 65–74 years old living in other rural counties who had the highest RSR of 96.9% (95% Cl, 82.9–99.5). However, Whites were considered to have a better RSR. This was also due to the statistical precision as mentioned above.

**Conclusion:**

There were significant racial differences in the RSRs of CerCancer. Overall, Black women experienced the worst RSRs compared to their White counterparts.

## Background

Cervical cancer (CerCancer) is the fourth most frequent cancer in women worldwide and ranks 14th in frequency in the US [1]. CerCancer remains a major cause of cancer-related mortality worldwide [2]. The CerCancer death rate in the US, in 2016 was 2.2 per 100,000 which was less than half of that in 1975 (5.6 per 100,000), this was due to declines in incidence and the early detection of the cancer through screening. From 2007 to 2016, the death rate decreased by about 1 % per year in women 50 years of age and older, but was stable in women under 50 years of age [3]. From 2005 to 2014, the CerCancer mortality rate in Alabama was 3.2 per 100,000 population, which was significantly, higher than the US rate of 2.3 per 100,000 population. Black Alabamians have a significantly higher CerCancer mortality rate than their White counterparts with a rate of 5.2 per 100,000 and 2.7 per 100,000 population respectively [4]. In the past 40 years and as a result of increased surveillance and improved treatments [5], both the incidence and mortality rates of CerCancer have significantly decreased. Although the decline in mortality from CerCancer has occurred across all racial and ethnic groups, a disproportionate burden of CerCancer still exists between Blacks and their White counterparts [6]. It has also been found that Blacks are more likely to be diagnosed with advanced stages of CerCancer compared to their White counterparts [7].

Survival of cancer patients in tandem with incidence, prevalence and mortality, constitute some of the fundamental basic indicators of cancer burden. Population-based survival of cancer patients is a valuable indicator as has been shown by Cancer registries for over 60 years [8]. The healthcare systems are usually inaccessible and not affordable for the underserved minority population, especially in the medical facilities with advanced equipment for cancer treatment. As usually presented by clinicians, however, it varies greatly in many ways for the survival of patient groups with a particular disease treated in individual hospitals [8]. It has been shown that cancer registries, which are population-based, have important roles to play both at national and international levels in the improvement of cancer patient’s care programs and policies. Monitoring of cancer trends, care patterns, survival estimates and provision of evidence-based outcomes for clinicians, public health administrators and policy makers are made possible due to data that is accurate and emanates from population-based cancer registries [9].

Survival measures are usually stratified into three major groups: Firstly, the overall survival group, which includes all causes of death; secondly, net survival group, where the competing causes of death are removed; and thirdly, the crude survival group, which consists of death resulting from other competing causes, death from the cancer, or surviving. Depending on whether cause of death information is available, both net survival and crude survival were calculated differently [9]. Of all the three groups, the overall survival is the most easily understood group. It is also the most reliable and accessible survival measure because it uses death from all causes as the endpoint. However, the overall survival is not detailed enough to provide information on survival associated with cancer diagnosis. Higher survival may be a result of fewer deaths from other causes or fewer deaths from the specific cancer [9].

Quantifying changes for cancer survival patients is significantly important, as efficacy of the advanced treatment therapies in clinical trials are of limited practical value since these treatment therapies cannot be applied into clinical practice. However, there are only two methodological challenges in assessing and tracing cancer survival trends in a specific population. Firstly, medical care has generally improved over time, but still it is difficult to determine that this improvement of survival of cancer patients is because of improvement of cancer treatment regimens or because of increased life expectancy in the population as a whole [10]. Secondly, because of some limitations in calculating cancer-specific survival as cause of death, information from death certificates are often not accurate. This may not reflect cancer-associated mortality in patients who die from complications and death due to cancer [11, 12].

The basic way of categorizing how far a cancer has spread from its site of origin, is determined by the Surveillance, Epidemiology, and End Results (SEER) summary stages. Most of the North America cancer registries reported their data by the summary stages. These staging categories are broad enough in measuring the success of both cancer control efforts and other epidemiologic efforts [13]. Whites compared to Blacks have been found to have higher survival rates of CerCance in several studies [14–19]. Many factors can be attributed to racial differences, lack of early detection and late stages at presentation [16, 20], no adherence to recommended follow-up care of cervical dysplasia [21], and socioeconomic status (SES) [22–25]. After controlling for CerCancer stage and treatment, however, a number of studies have not found racial differences in relative survival ratio estimates [26–28]. Due to these conflicting results, it has been emphasized there is a need to further examine the association between CerCancer relative survival ratios and race [16, 18, 20, 28]. Thus, the objective of this study was to assess racial differences in the 5-year relative survival ratios (RSR) of CerCancer by stage at diagnosis, between African American (Black) and White women living in Alabama, using SEER data from 2004 to 2013.

## Methods

### Study populations and data source

There were 24,543 White and Black women aged 15 or more years diagnosed with invasive CerCancer in the 18 SEER registries from 2004 to 2013. Of these 21,059 (85.8%) were White and 3484 (14.2%) were Black (Table 1). The SEER program of the National Cancer Institute (NCI) is an authoritative source of information on cancer incidence and survival in the US. The SEER program currently collects and publishes cancer incidence and survival data from population-based cancer registries covering approximately 30% of the US population. The SEER coverage is composed of 25% Whites, 26% African Americans, 38% Hispanics, 44% American Indians and Alaska Natives, 50% Asians, and 67% Hawaiian/Pacific Islanders; the SEER program is considered a benchmark for cancer survival surveillance in the US [29].

**Table 1:** 5-Year Relative Survival (age standardized) SEER 18, Malignant Derived SS 2000 (Localized, Regional, Distant and Unknown Stages) Cervical Cancer in Black and White Alabamians by County, Includes Cases Diagnosed in 2004–2013

County	Stage	Number of women alive at the start of the first interval	5-year % relative survival	The relative cumulative survival CIs lower & upper	Number of women alive at the start of the first interval	5-year % relative survival	The relative cumulative survival CIs lower & upper
		**Blacks**			**Whites**		
Urban	Localized	223	72.7	55.4–84.2	1647	77.8	70.7–83.3
	Regional	209	43.9	31.8–55.4	1204	48.8	43.5–53.9
	Distant	97	^a^	^a^	472	12.2	8.3–17.0
	Unknown	21	^a^	^a^	108	30.7	19.1–43.2
Black Belt	Localized	94	73.2	47.4–87.8	1088	83.8	74.5–89.9
	Regional	115	^a^	^a^	809	49.5	43.4–55.4
	Distant	36	^a^	^a^	283	14.2	9.0–20.6
	Unknown	14	17.0	13.8–20.5	100	40.7	28.7–52.3
Other Rural	Localized	1025	83.8	74.2–90.1	7323	83.7	79.9–86.8
	Regional	1089	49.0	43.4–54.3	5426	51.2	48.7–53.7
	Distant	408	11.7	7.4–17.0	1959	13.6	11.4–16.1
	Unknown	153	35.4	26.2–44.7	640	39.8	34.4–45.2

CIs Confidence interval: The level is 95%

^a^ The statistic could not be calculated (The RSR could not be calculated because there was only expected percentage and the observed percentage was missing)

### Variables

**Fig. 1:**
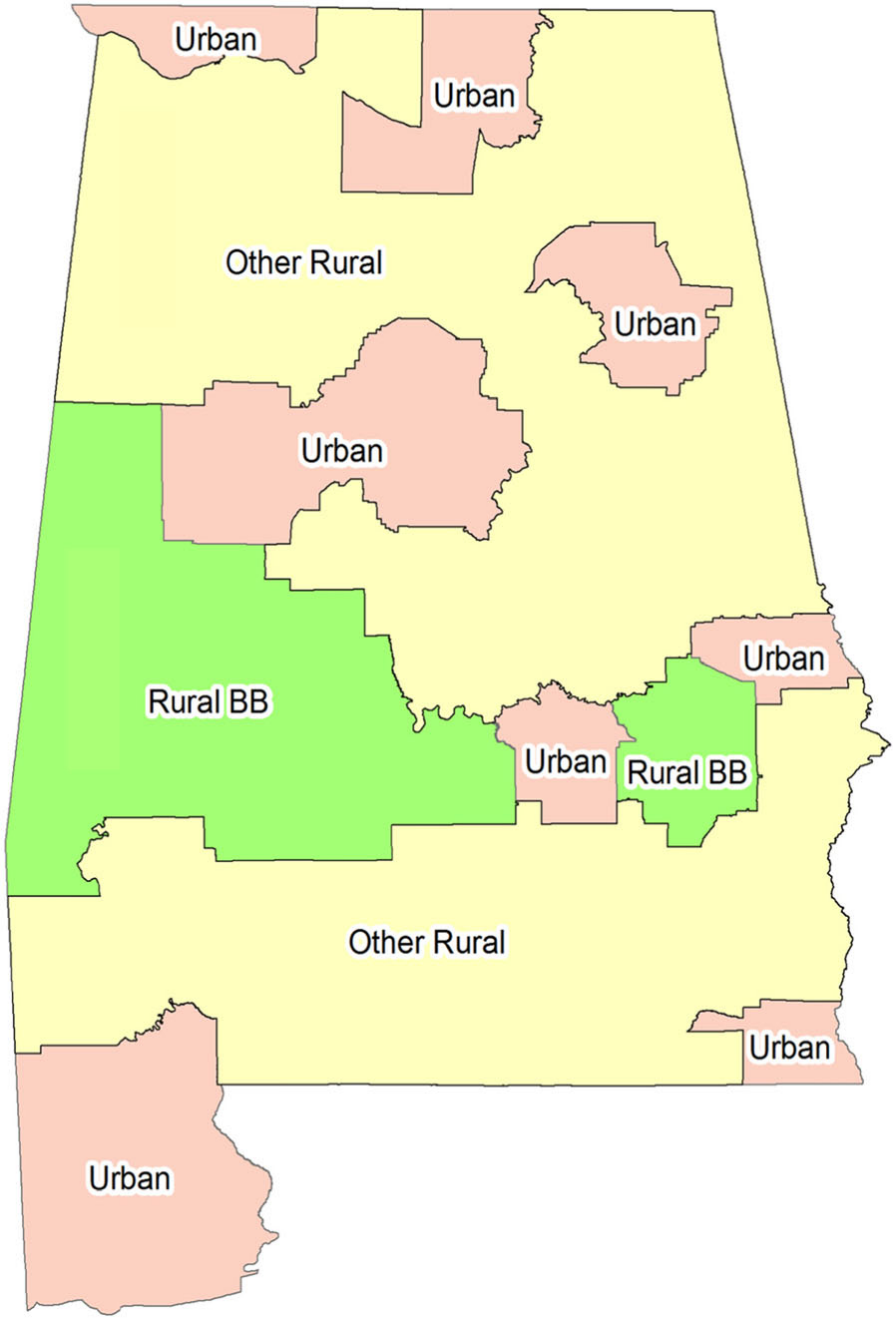
Urban, rural Black Belt and other rural counties of Alabama.  Urban Counties: Baldwin, Calhoun, Etowah, Houston, Jefferson, Lauderdale, Lee, Madison, Mobile, Montgomery, Morgan, Shelby, Tuscaloosa.  Rural Black Belt Counties: Bullock, Choctaw, Dallas, Greene, Hale, Lowndes, Macon, Marengo, Perry, Pickens, Sumter, Wilcox.  Other Rural Counties: Autauga, Barbour, Bibb, Blount, Butler, Chambers, Cherokee, Chilton, Clarke, Clay, Cleburne, Coffee, Colbert, Conecuh, Coosa, Covington, Crenshaw, Cullman, Dale, DeKalb, Elmore, Escambia, Fayette, Franklin, Geneva, Henry, Jackson, Lamar, Lawrence, Limestone, Marion, Marshall, Monroe, Pike, Randolph, Russell, St. Clair, Talladega, Tallapoosa, Walker, Washington, Winston

Patients who were diagnosed with primary malignant localized, regional, distant and unknown CerCancer between 2004 and 2013 were selected from the November 2015 submission of SEER-18 registries, including the three groups of counties (urban, Black Belt and other rural counties) of Alabama (Fig. 1). Patients were followed for 5 years from diagnosis or until December 2013. Data for the patients who had died from CerCancer their diagnosis was confirmed by death certificates or by autopsy [29].

The SEER*Stat software provides data on age standard adult cancer populations (ages 15 years and older) to calculate age standardized survival. The International age-standardized cancer survival was used to compare survival across time or different cancer populations with different age distributions [30]. The SEER*Stat software provides weights by 5-year age groups using the age variable, “Age recode with less than one year old”, and by five age groups (15–44, 45–54, 55–64, 65–74, 75 years and older) as defined by Corazziari et al. in 2004, weights for ages under 15 is zero [30]. Therefore, the appropriate age standard at diagnosis CerCancer survival that was classified into above mentioned five groups was used in this study.

The Site, Morphology and Site recodes International Classification of Diseases for Oncology (ICD-O)-3/ World Health Organization (WHO) 2008, where cancer sites are categorized were used to select the cancer site of our interest “cervix uteri.” In SEER*Stat software there are two variables which have the same name “stage”. The variables with definitions stage I, stage II, stage III and stage IV are based on the American Joint Committee on Cancer (AJCC) staging scheme. The stage variable with definitions local, regional, distant and unknown uses the Summary Stage algorithm. Both stages use the extent of disease information (size of tumor, extension of tumor and associated lymph node status) to calculate the stage variable. The summary stage tends to be used when doing analysis over time, as there is more consistency over time. In this study our data were stratified by SEER historic staging scheme at diagnosis, provided information for in situ and invasive CerCancer, with the invasive CerCancer being classified into the following four stage categories: (i) localized (invasive cancer confined to the organ of origin), (ii) regional (spread to adjacent organs and/or regional lymph nodes by direct extension), (iii) distant (extension to organs other than those covered in the regional category or metastases to distant organs or distant lymph nodes), and (iv) unknown (cancer of unknown primary origin), these four stages are critical and strong predictors of cancer survival [29].

### Statistical analysis

Relative cancer survival is cancer survival in the absence of other causes of death and is usually calculated using some demographic factors such as some socioeconomic status (SES), geographic location, race and annual life tables. These life tables are recommended when using statistics that are limited from 1992 to 2013, or for limited geographic locations, race and ethnicity groups other than Whites and Blacks [31]. Relative survival is a net survival measure representing cancer survival in the absence of other causes of death. Relative survival is defined as the ratio of the proportion of observed survivors in a cohort of cancer patients to the proportion of expected survivors in a comparable set of cancer free individuals. On the other hand, cause-specific survival is considered a net measure survival because it represents survival of a specified cause of death in the absence of other causes of death [32]. In the absence of life tables or are inaccurately estimate expected survival for a specific group of cancer patients, cause-specific survival considered more accurate methods than relative survival [33]. In this study, the five-year relative survival of CerCancer was used as it is the most suitable method to compare survival between different registries worldwide. This is because cause of death may not be easily available or there may be inconsistency in the accurate determination of cause of death globally [34]. However, when accurate life tables are available, relative survival will only be calculated to represent expected survival of the cohort of cancer patients. In population studies, there is incomparability of the observed survival between different populations and the availability of life tables of different general populations such as those for Blacks and Whites. In this study, five-year relative survival methods were the most appropriate methods to use for the estimation of the net cancer survival, rather than the cause-specific survival, whereas the net cancer survival can be estimated by other relative survival methods, rather than by the cause-specific survival [8]. Additionally, a RSR of 100% implies that a cancer patient cohort is just as likely to survive the given interval as a cohort in the general population of the same sex and age; it does not necessarily mean that everyone in the group has survived cancer [29].

The analysis was conducted using the SEER*Stat software version 8.3.2 developed by the National Cancer Institute (NCI) for the analysis and reporting of cancer statistics, in particular, statistics regarding CerCancer survival, in conjunction with Excel functions to model and analyze the racial dynamics of CerCancer survival. Calculated statistics such as relative and Confidence Intervals (CI) were obtained and presented in Microsoft word 2016 tables and bar graphs using Excel functions. When comparing two RSRs for two different or independent populations (Blacks and Whites stratified by age group, county and stage of CerCancer) to determine whether a significant difference exists between them, then the 95% CIs for both rates are compared.

A confidence interval provides a range of plausible values (lower and upper bounds) it provides a range of values, which is likely to contain the population parameter of interest. In this study, we calculated the level of confidence set at 95%. Confidence intervals use the variability of data to assess the precision or accuracy of the estimated statistics. Confidence intervals were used to compare two groups (Blacks and Whites). The precision of the statistics depends on some factors which affect the width of the confidence interval these include the size of the sample, the confidence level, and the variability in the sample. A tighter confidence interval with a smaller margin of error will be because of a larger sample size or lower variability. Conversely, a wider confidence interval with a larger margin of error will be because of a smaller sample size or a higher variability. A higher level of confidence will tend to produce a broader confidence interval. A close-fitting interval at 95% or higher confidence is ideal. The confidence interval can be described as the difference between the upper and lower bounds. The confidence interval around the mean is twice the margin of error. The confidence interval is half the width of the margin of error [35].

## Results

**Fig. 2:**
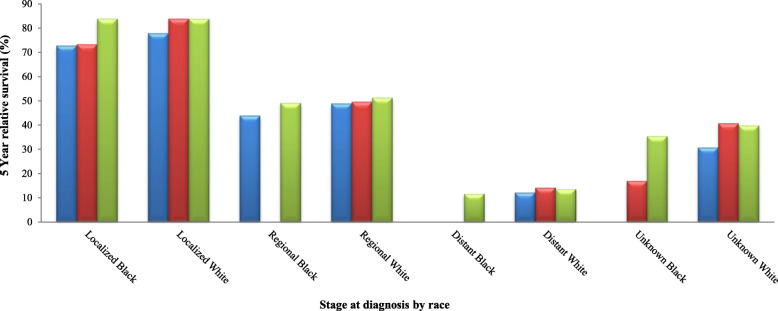
5-year relative survival rates of cervical cancer in Blacks and Whites in Alabama.  Urban.  Black Belt.  Other Rural

From 2004 to 2013, in the 18 SEER registries there were 24,543 White and Black women aged ≥15 years that were diagnosed with invasive CerCancer. Of these 21,059 (85.8%) were Whites and 3484 (14.2%) were Blacks. As presented in Table 1 and Fig. 2, we compared the highest 5 year Relative Survival Rates (RSRs) between Blacks and Whites living in all the three county groups (urban, BB and other rural counties) of Alabama. It was observed that in general Whites diagnosed with localized stages of CerCancer and living in all the three county groups always had higher RSRs, compared to their Black counterparts. The only exception was in Blacks living in the other rural counties and in Whites living in BBC who had the same overall highest RSRs. In the urban counties, Whites diagnosed with localized stage of CerCancer, had a significantly higher RSR of 77.8% (95% CI 70.7–83.3%), compared to Blacks who had a lower RSR of 72.7% (95% Cl, 55.4–84.2). Whites living in the BBC and Blacks living in the other rural counties diagnosed with localized stage of CerCancer had the same significantly highest RSRs of 83.8% (95% CI 74.5–89.9) and 83.8% (95% Cl, 74.2–90.1) respectively. Whites diagnosed with localized stage of CerCancer living in the BBC had the significantly highest RSR of 83.8% (95% CI 74.5–89.9) compared to Blacks who had an RSR of 73.2% (95% Cl, 47.4–87.8). Although in other rural counties, Blacks diagnosed with localized stage of CerCancer had a slightly higher RSR of 83.8% (95% Cl, 74.2–90.1), compared to Whites who had an RSR of 83.7% (95% CI 79.9–86.8%), Whites were considered to have a better RSR. This was because of the precision of the statistics, which depended on their sample size and variability. Whites had a larger sample size of 7323 women and a lower variability therefore, more reliability resulting in a tighter confidence interval (86.8–79.9 = 6.9) with a smaller margin of error (6.9/2 = ± 3.45%). This was compared to the smaller sample size of 1025 women and a higher variability therefore, less reliability in Blacks resulting in a wider confidence interval (90.1–74.2 = 15.9) with a larger margin of error (15.9/2 = ± 7.95%), see Table 1.

**Fig. 3:**
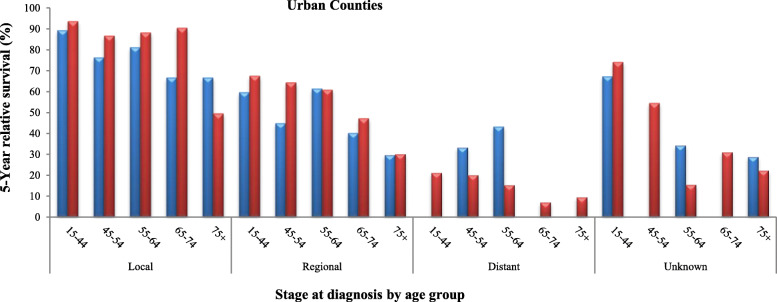
5-year relative survival rates of cervical cancer in Blacks and Whites in urban counties.  Blacks.  Whites

**Fig. 4:**
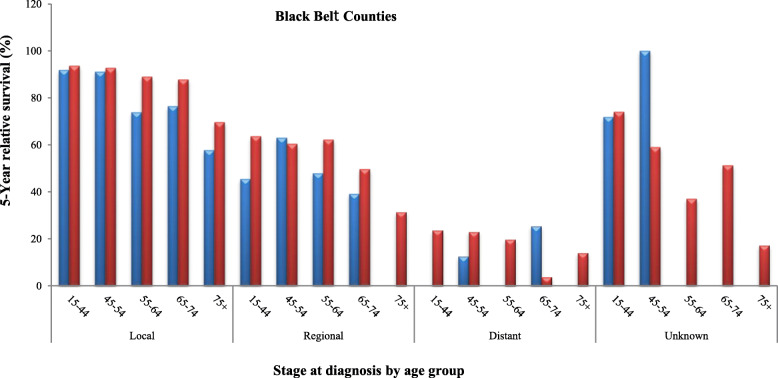
5-year relative survival rates of cervical cancer in Blacks and Whites in Black Belt counties.  Blacks.  Whites

**Fig. 5:**
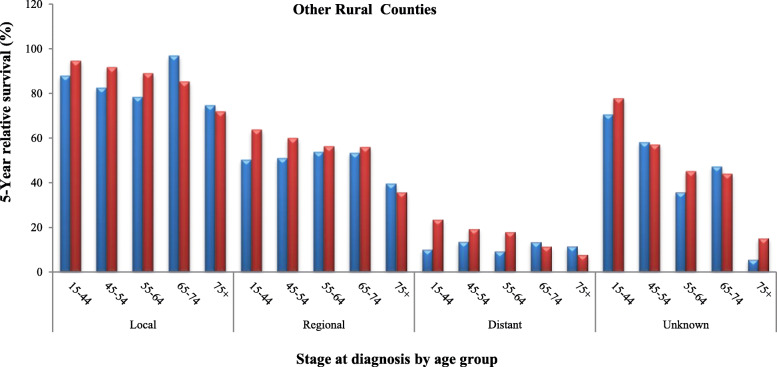
5-year relative survival rates of cervical cancer in Blacks and Whites in other rural counties.  Blacks.  Whites

Tables 2, 3 and 4 and Figs. 3, 4 and 5 represent detailed comparisons of the RSRs between Blacks and Whites living in the urban, BB and other rural counties of Alabama stratified by five age groups (15–44, 45–54, 55–64, 65–74, and 75 years and older) and four stages of CervCancer (localized, regional, distant and unknown). It was observed that young Whites 15–44 years old, diagnosed with localized stage of CerCancer and living in all the three county groups always had the highest RSRs, compared to their Black counterparts. The only exception was in Blacks 65–74 years old, living in the other rural counties who had the highest RSR. In the urban counties, Whites diagnosed with localized stage of CerCancer, had a significantly higher RSR of 93.6% (95% CI 91.4–95.2%), compared to Blacks who had an RSR of 89.2% (95% Cl, 79.6–94.5). In addition, Whites diagnosed with localized stage of CerCancer living in the BBC had a significantly higher RSR of 93.6% (95% CI 90.8–95.6), compared to Blacks who had an RSR of 91.8% (95% Cl, 75.0–97.5). However, in the other rural counties, Blacks diagnosed with localized stage of CerCancer had the highest RSR of 96.9% (95% Cl, 82.9–99.5), compared to Whites who had an RSR of 94.6% (95% CI 93.6–95.4%). Although, Blacks diagnosed with localized stage of CerCancer and living in other rural counties had a higher RSR, compared to their White counterparts, their RSR was better. As previously stated in Table 1 and Fig. 2 this is because of the precision of the statistics, which depended on the sample size and variability. Whites had a larger sample size of 4105 women and a lower variability therefore, more reliability resulting in a tighter confidence interval (95.4–93.6 = 1.8) with a smaller margin of error (1.8/2 = ± 0.9%). This was compared to the smaller sample size of 78 women and a higher variability therefore, less reliability resulting in Blacks resulting in a wider confidence interval (99.5–82.9 = 16.6) with a larger margin of error (16.6/2 = ± 8.3%), see Table 4.

**Table 2:** 5-Year Relative Survival (age standardized) SEER 18, Malignant Derived SS 2000 Cervical Cancer in Black and White Alabamians, Living in Urban by Age Includes Cases Diagnosed in 2004–2013

Stage	Age group in years	Number of women alive at the start of the first interval	5-year % relative survival	The relative cumulative survival CIs lower & upper	Number of women alive at the start of the first interval	5-year % relative survival	The relative cumulative survival CIs lower & upper
		**Blacks**			**Whites**		
Localized	15–44	108	89.2^a^	79.6–94.5^a^	954	93.6^a^	91.4–95.2^a^
	45–54	54	76.2^a^	58.2–87.2^a^	341	86.6^a^	81.4–90.5^a^
	55–64	30	81.1^a^	55.5–92.8^a^	185	88.1^a^	79.7–93.1^a^
	65–74	14	66.6^a^	26.9–88.2^a^	109	90.4^a^	76.4–96.3^a^
	75^b^	17	66.6^a^	23.4–89.2^a^	58	49.5^a^	29.8–66.5^a^
Regional	15–44	64	59.6^a^	42.7–73.0^a^	388	67.4	61.6–72.6
	45–54	52	44.9^a^	28.4–60.2^a^	329	64.3^a^	57.9–70.0^a^
	55–64	37	61.3^a^	39.6–77.2^a^	247	60.8^a^	53.0–67.6^a^
	65–74	26	40.2^a^	16.1–63.4^a^	148	47.2^a^	36.3–57.4^a^
	75^b^	30	29.7^a^	7.3–56.9^a^	92	30.1^a^	17.9–43.3^a^
Distant	15–44	23	^b^	^b^	103	21.2^a^	12.4–31.6^a^
	45–54	25	33.3^a^	14.6–53.2#	125	20.1^a^	12.1–29.7^a^
	55–64	20	43.2^a^	19.2–65.3^a^	112	15.3#	7.9–24.8^a^
	65–74	17	0.0	^b^	76	7.1^a^	1.4–19.3^a^
	75^b^	12	0.0	^b^	56	9.5^a^	3.0–20.7^a^
Unknown	15–44	7	67.2^a^	19.2–90.9^a^	39	74.0^a^	53.9–86.4^a^
	45–54	4	0.0	^b^	18	54.4^a^	28.9–74.3^a^
	55–64	3	34.3^a^	0.8–78.7^a^	15	15.5^a^	0.9–47.6^a^
	65–74	3	^b^	^b^	11	31.0^a^	7.3–59.2^a^
	75^b^	4	28.8^a^	0.8–72.7^a^	25	22.3^a^	6.6–43.7^a^

CIs Confidence interval: The level is 95%

^a^ The relative cumulative survival increased from a prior interval and has been adjusted

^b^ The statistic could not be calculated (The RSR could not be calculated because there was only expected percentage and the observed percentage was missing)

**Table 3:** 5-Year Survival (age standardized) SEER 18, Malignant Derived SS 2000 Cervical Cancer in Black and White Alabamians, Living in Black Belt Counties Includes Cases Diagnosed in 2004 2013

Stage	Age group in years	Number of women alive at the start of the first interval	5-year % relative survival	The relative cumulative survival CIs lower & upper	Number of women alive at the start of the first interval	5-year % relative survival	The relative cumulative survival CIs lower & upper
		**Blacks**			**Whites**		
Localized	15–44	42	91.8^b^	75.0–97.5^b^	645	93.6^b^	90.8–95.6^b^
	45–54	15	91.1^b^	42.4–99.0^ba^	230	92.7^b^	86.9–95.9^b^
	55–64	24	73.8^b^	47.3–88.4^b^	113	89.0	76.9–95.0
	65–74	9	76.4^b^	23.2–95.2^b^	65	87.8^b^	67.1–95.9^b^
	75^c^	4	57.8^b^	3.8–91.2^b^	35	69.6^b^	42.7–85.7^b^
Regional	15–44	32	45.5^b^	23.4–65.3^b^	283	63.6^b^	56.6–69.7^b^
	45–54	26	63.0^b^	37.4–80.5^b^	199	60.4^b^	51.5–68.2^b^
	55–64	20	47.9^b^	22.1–69.9^b^	153	62.2^b^	52.1–70.7^b^
	65–74	27	39.2^b^	15.1–62.9^b^	92	49.7	35.7–62.2
	75^c^	10	^c^		82	31.5^b^	18.8–44.9^b^
Distant	15–44	5	0.0	^c^	71	23.7^b^	13.8–35.6^b^
	45–54	8	12.6^b^	0.7–42.6^b^	83	23.1^b^	8.8–33.7^b^
	55–64	12	^c^	^c^	68	19.8^b^	0.3–33.9^b^
	65–74	8	25.5^b^	3.7–56.7^b^	37	3.9^b^	3.5–16.4^b^
	75^c^	3	0.0	^c^	24	14.1^b^	13.8–31.9^b^
Unknown	15–44	5	71.8^b^	8.7–95.6^b^	30	74.0^b^	49.3–88.0^b^
	45–54	2	100.0*^b^	^bc^	22	59.0^b^	33.5–77.5^b^
	55–64	3	0.0	^c^	17	37.1^b^	13.3–61.4^b^
	65–74	1	0.0	^c^	16	51.3^b^	23.1–73.7^b^
	75^c^	3	0.0	^c^	15	17.3^b^	2.9–42.1^b^

CIs Confidence interval: The level is 95%

^a^ The width of the confidence interval is more than 25% larger than if the normal approximation was applied

^b^ The relative cumulative survival increased from a prior interval and has been adjusted

^c^ The statistic could not be calculated (The RSR could not be calculated because there was only expected percentage and the observed percentage was missing)

**Table 4:** 5-Year Relative Survival (age standardized) SEER 18, Malignant Derived SS 2000 Cervical Cancer in Black and White Alabamians, Living in Other Rural Includes Cases Diagnosed in 2004–2013

Stage	Age group in years	Number of women alive at the start of the first interval	5-year % relative survival	The relative cumulative survival CIs lower & upper	Number of women alive at the start of the first interval	5-year % relative survival	The relative cumulative survival CIs lower & upper
		**Blacks**			**Whites**		
Localized	15–44	508	87.9^b^	84.1–90.8^b^	4105	94.6^b^	93.6–95.4^b^
	45–54	241	82.5^b^	75.2–87.8^b^	1669	91.7	89.7–93.3
	55–64	146	78.3^b^	67.8–85.7^b^	867	89.0	85.7–91.5
	65–74	78	96.9*^b^	82.9–99.5^ba^	449	85.3^b^	79.3–89.7^b^
	75^c^	52	74.6^b^	39.6–91.1^b^	233	71.8^b^	59.8–80.8^b^
Regional	15–44	356	50.3^b^	44.3–56.0^b^	1742	63.7	60.9–66.3
	45–54	286	51.0^b^	44.0–57.5^b^	1420	59.9	56.7–62.9
	55–64	209	53.8	44.6–62.0	1092	56.3^b^	52.6–59.9^b^
	65–74	115	53.4^b^	40.6–64.7^b^	674	56.0	51.1–60.6
	75^c^	123	39.6^b^	27.7–51.3^b^	498	35.8^b^	29.5–42.1^b^
Distant	15–44	92	10.1^b^	4.1–19.2^b^	453	23.5^b^	19.2–28.2^b^
	45–54	106	13.6	6.7–22.9	490	19.3^b^	15.3–23.8^b^
	55–64	100	9.3^b^	4.1–17.1^b^	522	17.9	14.1–22.2
	65–74	59	13.4^b^	5.0–25.9^b^	286	11.4^b^	6.9–17.3^b^
	75^c^	51	11.5^b^	3.4–24.9^b^	208	7.7^b^	3.7–13.7^b^
Unknown	15–44	52	70.5^b^	54.1–81.9^b^	218	77.7^b^	70.1–83.5^b^
	45–54	24	58.1^b^	34.8–75.7^b^	125	57.1^b^	46.8–66.1^b^
	55–64	25	35.8^b^	16.1–56.1^b^	92	45.2^b^	32.8–56.8^b^
	65–74	21	47.2^b^	23.1–68.0^b^	87	44.0^b^	31.6–55.7^b^
	75^c^	31	5.6^b^	0.4–22.4^b^	118	15.1^b^	7.5–25.3^b^

CIs Confidence interval: The level is 95%

^a^ The width of the confidence interval is more than 25% larger than if the normal approximation was applied

^b^ The relative cumulative survival increased from a prior interval and has been adjusted

^c^ The statistic could not be calculated (The RSR could not be calculated because there was only expected percentage and the observed percentage was missing)

In this study, we interpret our results of confidence intervals overlap based on the Cornell Statistical Consulting Unit, which states that determining whether confidence intervals overlap is an overly conservative approach for identifying significant differences between groups. It is true that when confidence intervals do not overlap, the difference between groups is statistically significant (at the 0.05 level of significance). However, when there is some overlap, the difference might still be significant (Cornell Statistical Consulting Unit, 2008). Blacks living in the BBC, aged 45 to 54 years and diagnosed with unknown stage of CerCancer had 100% survival rate, which indicated that all the patients survived. This result was excluded in this study because of a very small sample size. There were only two women alive at the start of the first interval. It should be noted that Blacks diagnosed with regional, distant and unknown stages of CerCancer and living in urban and BB counties their survival rates could not be determined because of insufficient data (Their RSRs could not be calculated because there was only the expected percentage and the observed percentage was missing).

## Discussion

This study was conducted to assess the racial differences in the 5-year Relative Survival Rates (RSRs) of CerCancer by stage at diagnosis between Black and White women, living in the urban, BB and other rural counties of Alabama. The study results indicate that there are significant racial differences in the RSRs. However, examination of the characteristics (age at diagnosis, stages of the CerCancer, and geographical locations) between Blacks and Whites showed that these two groups of women were dissimilar in these characteristics. Comparing ostensibly dissimilar groups remains a challenging aspect of epidemiological studies.

In a previous study, the RSR for all women with CerCancer was 66%. However, survival rates can vary by factors such as race, ethnicity and age. The RSRs for White women was 69% and for Black women was 56%. For White women under 50 years, the RSR was 78%. For Black women 50 years and older, their RSR was 47% [36]. The RSRs also depend on the diagnosed stage of CerCancer. When detected at an early stage, the RSR for women with invasive CerCancer was 92%. About 44% of women with CerCancer was diagnosed at an early stage. If CerCancer has spread to surrounding tissues or organs and/or the regional lymph nodes, the RSR was 56%. If the cancer has spread to a distant part of the body, the RSR was 17% [36].

Our analysis showed that there were racial differences in the 5-year relative survival rate (RSRs) of CerCancer. Overall, Black women experienced the worst RSRs compared to their White counterparts. This study found that Whites diagnosed with localized stages of CerCancer and living in all the three county groups always had better chances of survival. This was because their RSRs were always more than 77%, compared to their Black counterparts. The only exception was in Blacks living in the other rural counties, who had significantly highest RSR of 83.8%, which was the same as in Whites living in the BBC. Although, Whites living in the other rural counties had a slightly lower RSR of 83.7%, compared to both Blacks and Whites with a slightly higher RSR of 83.8%, their RSR was better. This was because of the precision of the statistics, which depended on their larger sample size and a lower variability therefore, more reliability resulting in a tighter confidence interval with a smaller margin of error.

This study also highlights the comparisons of the RSRs for both Blacks and Whites by CerCancer stages (localized, regional, distant and unknown), age groups (15–44, 45–54, 55–64, 65–74, and 75 years and older) and living in the urban, BB and other rural counties of Alabama. In all the three groups of counties, the study results indicate that Whites 15–44 years old diagnosed with localized stage of CerCancer had a higher RSR of 93.6% for those living in both the urban and BB counties, and the RSR of 94.6% for those living in the other rural counties. The only exception was in Blacks 65–74 years old and living in the other rural counties who had the highest RSR of 96.9%. However, Whites were considered to have a better RSR. As previously stated, this was because of the precision of the statistics, which depended on their larger sample size and a lower variability therefore, more reliability resulting in a tighter confidence interval with a smaller margin of error.

Age is known to be an important prognostic factor for developing CerCancer. It has been found that the RSR is highly dependent on the age at diagnosis, which was in agreement with the findings from the European Alcohol Policy Alliance (EUROCARE) Study conducted in the European countries [37] as well as the results reported by Adami et al. [38]. Additionally, in this study we compared the RSRs not only by race but also by age groups, stages of CerCancer and geographical locations. Similarly, our results are in agreement with the findings from the above previous studies [37, 38] regarding the age. This was observed to be mostly true in Whites, especially those living in other rural counties, diagnosed with localized, regional, distant and unknown stages, their RSRs for CerCancer gradually declined with increasing age. Overall, in this study, both Blacks and Whites diagnosed with CerCancer in all its stages living in the urban, BB and other rural counties, it was observed that as the age increased the chances of surviving CerCancer 5 years after diagnosis decreased. This was irrespective of the race, stages of CerCancer and geographical locations with few exceptional cases.

The results of this study are in agreement with what Hicks et al. reported in their findings there was clearly, a notable disparity in CerCancer survival between various minority populations and Whites [7]. In our recent study [7], the results were similar to those of Hicks et al., whereby it was found that, identifiable factors that affect survival disparity were inadequate screening and/or treatments, inappropriate treatments or comorbid illnesses [7]. In addition, according to the National Health Survey, similar CerCancer screening rates were reported among Black and White Americans [39]. Significantly, in contrast, our recent study findings [40] regarding CerCancer screening, showed high rates of CerCancer mortality, which was more than triple in Black Alabamians (between 2002 and 2012), despite their high screening rates (between 2000 and 2010), compared to their White counterparts. This study shows conclusively that in Alabama, a disparity still exists for the high CerCancer mortality in Blacks, despite the higher rates of screening as would otherwise be expected [40]. In spite of all this, Blacks were still more likely to be diagnosed with advanced stages of CerCancer, and their chances of surviving 5 years after diagnosis was lower compared to their White counterparts. The racial differences in CerCancer stage at diagnosis may be due to differences in the quality of screening and follow-up after abnormal Pap test results [39]. Access to quality health care was often compromised among underserved minorities, particularly Blacks, the uninsured and older women [7, 41].

In this study, we had some limitations in our analysis. Although various prognostic factors were available for many States in the SEER database and in other tumor registry databases, Alabama factors were not fully accessible in the databases. This is why these factors were not taken into consideration in the analysis. These factors included information on life-style or other individual factors such as screening access, socioeconomic status (SES) and comorbidities. Despite these limitations, the differences in CerCancer survival clearly highlight the importance of affordability and accessibility to and use of diagnostic and treatment facilities for minority populations. Differences in CerCancer survival and making adequate facilities affordable and accessible should be given priority. To reduce and/or eliminate the racial differences in CerCancer stages at diagnosis. Emphasis should also be given to promoting early diagnosis through quality screening, follow-up after abnormal Pap test results and access to quality health care, particularly in Blacks, the uninsured and older women living in Alabama, especially in the BBC.

## Conclusion

Although the gap in the 5-year relative survival rates (RSR) of CerCancer in Alabama has narrowed over time, the disparity still persists. If the dismal narrowing is sustained, it will reduce the racial disparities in CerCancer survival though at a very slow rate. Therefore, public health officials should monitor progress towards reduction and/or elimination of these disparities. Although further progress seems to be very slow given the current screening programs and treatments, it might be achieved by extending the quality of the screening programs. This could possibly be achieved by using more advanced screening tests. Follow-up after abnormal Pap test results or advances in treatments and access to quality health care, is often compromised among underserved minorities, particularly in older Black women, who happen to be mostly uninsured and living especially in the BBC of Alabama.

Overall, this study indicates that White women diagnosed with early stages of CerCancer living in the urban, BB and other rural counties, were more likely to survive compared to their Black counterparts. Confounder factors such as race and ethnicity, and biologic characteristics that need further exploration may influence reasons for these differences. The findings in this study indicate that Blacks diagnosed with distant stage of CerCancer especially those who are 65 years and older are less likely to survive 5 years after diagnosis with this stage of CerCancer. This highlights the need to investigate the genetic etiology, as well as the timing of diagnosis, treatment efficacy, and participation in clinical trials.

## Data Availability

**Data Source** Cervical cancer data are public available data collected by the Surveillance, Epidemiology and End Results (SEER) database for the years 2004 and 2013 were extracted and used in this study. The SEER program of the National Cancer Institute (NCI) is an authoritative source of information on cancer incidence and survival in the US. The SEER program currently collects and publishes cancer incidence and survival data from population-based cancer registries covering approximately 30% of the US population.

## Abbreviations

RSR: 5-year relative survival ratios
CerCancer: Cervical Cancer
SEER: Surveillance, Epidemiology, and End Results
CI: Confidence Intervals
BBC: Black Belt County
US: United States
HPV: human papilloma virus
Pap test: Papanicolaou test
NCI: National Cancer Institute
PC: Personal Computer
ICD-O: International Classification of Diseases for Oncology
WHO: World Health Organization
AJCC: American Joint Committee on Cancer
SR (t): Survival rate
SO (t): Observed survival
SP (t): Expected survival
EUROCARE: European Alcohol Policy Alliance
SES: Socioeconomic Status
TU CBR/RCMI: Tuskegee University Center for Biomedical Research/Research Centers in Minority Institutions
NIH: National Institute of Health
